# HIV-1 Protease Dimerization Dynamics Reveals a Transient Druggable Binding Pocket at the Interface

**DOI:** 10.1038/srep18555

**Published:** 2015-12-22

**Authors:** Fabio Pietrucci, Attilio Vittorio Vargiu, Agata Kranjc

**Affiliations:** 1 Sorbonne Universités, UPMC University Paris 6, CNRS - UMR 7590, IMPMC, F-75005 Paris, France; 2 Department of Physics, University of Cagliari, I-09042 Monserrato, Italy; 3 School of Pharmaceutical Sciences, University of Geneva, CH-1211 Geneva, Switzerland

## Abstract

The binding mechanism of HIV-1 protease monomers leading to the catalytically competent dimeric enzyme has been investigated by means of state-of-the-art atomistic simulations. The emerging picture allows a deeper understanding of experimental observations and reveals that water molecules trapped at the interface have an important role in slowing down the kinetics of the association process. Unexpectedly, a cryptic binding pocket is identified at the interface of the complex, corresponding to a partially bound dimer that lacks enzymatic function. The pocket has a transient nature with a lifetime longer than 1 μs, and it displays very favorable druggability features. Docking as well as MM-GBSA free-energy calculations further support the possibility to target the new binding site by means of inhibitors able to prevent the complete dimerization by capturing the inactive conformation. This discovery could open the way to the rational design of a new class of anti-HIV drugs.

Among current anti-AIDS treatments a major role is played by a set of competitive inhibitors binding to the active catalytic site of Human Immunodeficiency Virus type 1 (HIV-1) protease. This enzyme plays a crucial role in the maturation of the virus, by cleaving the Gag and Gag-Pol polyproteins at specific sites to produce functional proteins. The development of the protease inhibitors in the last 20 years is a success story of structure-based drug design^1^,^2^, rendering AIDS a chronic disease for patients with access to treatments. However, there is an active quest for better compounds, due to the toxicity of current drugs and to the insurgence of drug resistance through a pattern of mutations that reduce inhibitor affinity while rescuing viral fitness.

In the struggle to enlarge the repertoire of pharmaceutical targets and boost the development of novel drugs, a recent promising direction is to seek molecules interfering with protein-protein interactions, e.g., targeting interfaces of complexes^3^. Unfortunately, the search is often hampered by the lack of deep binding pockets at the interface, as deduced from crystallographic structures. On the other hand, molecular dynamics simulations, being able to capture conformational fluctuations of the biomolecules at finite temperature, can substantially enrich the picture provided by crystallography^4^,^5^. Examples are the discovery of a cryptic binding site in HIV integrase^6^, that led to the first FDA approved drug for this target^7^, and the identification of a transiently open pocket in tumor suppressor p53^8^. In this work, by investigating with molecular dynamics part of the dimerization mechanism of HIV-1 protease, we discover a cryptic transient binding site at the interface of the complex that displays favorable druggability features, what we hope will contribute to design a new class of inhibitors. Our results are compatible with nuclear spin relaxation experiments showing chemical exchange to occur on the μs-ms time scale at the residues that form the transiently opening pocket^9^,^10^.

HIV-1 protease is a homo-dimeric enzyme, and its protein-protein interface is formed by the three regions in Fig. 1: ordered by increasing contribution to the binding free energy^11^,^12^, they involve the flap tips, the core of the complex including active site catalytic triads (Asp25-Thr26-Gly27) and several hydrophobic contacts, and the four-stranded beta sheet formed by N- and C-termini of both monomers. The last years have seen several attempts to develop dimerization inhibitors. Typically, the proposed molecules target either the terminal beta sheet, by analogy with drugs interfering with amyloid aggregates, or they bind to the isolated monomer^2^,^13^,^14^,^15^. Intriguingly, the clinically employed drugs darunavir and tipranavir besides binding to the catalytic pocket displayed additional activity as dimerization inhibitors^16^, but the corresponding mechanism is the object of debate^12^,^17^,^18^,^19^,^20^. As we show in the following, a combination of docking, molecular dynamics, and MM-GBSA calculations suggests that the latter inhibitors could also bind to the newly discovered interface cavity of the protease dimer with an affinity comparable to the one for the catalytic pocket. Clearly, the relatively low estimated population of the secondary binding mode, together with the imperfect accuracy of state-of-the-art force fields and free energy calculation approaches, points to the necessity of further experimental investigations to compellingly assign the structural origin of the dimerization inhibition effect of the latter drugs. Nevertheless, our results encourage to search a new class of improved dimerization inhibitors against HIV-1 protease, and they suggest that similar mis-bound conformations could be present in other protein complexes, enlarging the repertoire of pharmaceutical targets.

## Results

### The free energy landscape reveals a step-wise opening of the dimer

We studied the detailed dimerization mechanism of HIV-1 (subtype B) protease with molecular dynamics simulations explicitly including all protein atoms and water molecules. To reconstruct free energy landscapes as a function of different order parameters we employed an enhanced sampling method, bias exchange metadynamics^21^, complemented by the weighted histogram analysis method (WHAM)^22^. We also employed standard (unbiased) MD simulations to confirm the results and estimate the lifetime of the new metastable cavity. Simulations sum up to a total time of about four microseconds (all details are provided in the Methods section).

We started the bias exchange simulations from a crystallographic HIV-1 protease structure^23^. The trajectories sample multiple “opening” events, characterized by the breaking of interatomic contacts at the protein-protein interface and penetration of water at the level of flap tips, catalytic triads, and hydrophobic region. The opening of the complex is also evident from increase in the distance between centers of mass of the monomers, passing from 2.7 nm to values larger than 3.2 nm. The interdigitated N- and C-terminal beta sheet is more reluctant to break and has a strong tendency to keep its native conformation, thus hampering a full dissociation of the complex. Eventually, after the loss of all backbone hydrogen bonds in the beta sheet, the complete dissociation of the two monomers is observed.

**Figure 2:**
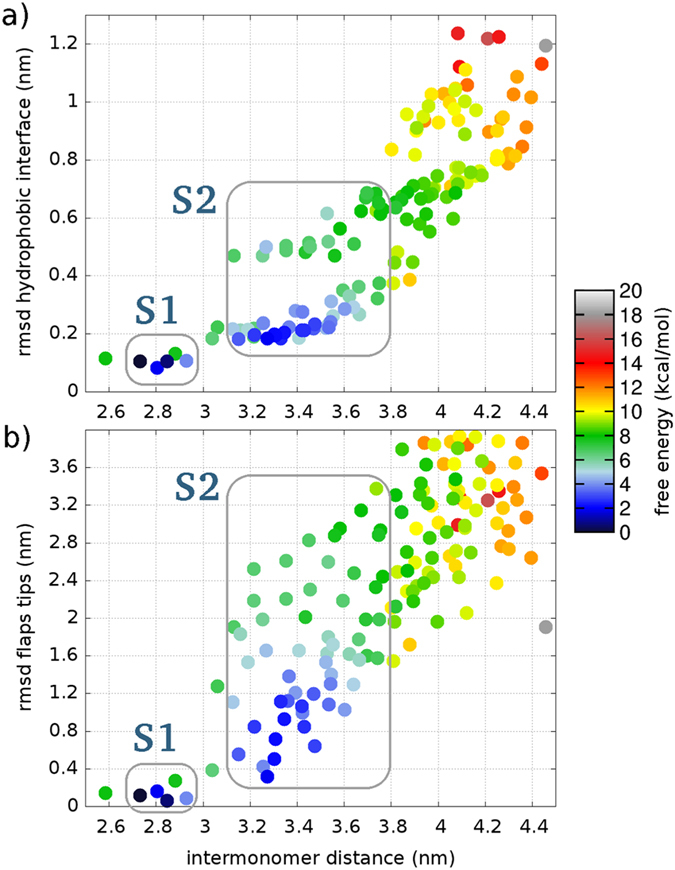
Free energy landscape as a function of different collective variables. The 150 structural clusters composing the ensemble of conformations are shown as colored dots.

**Figure 3:**
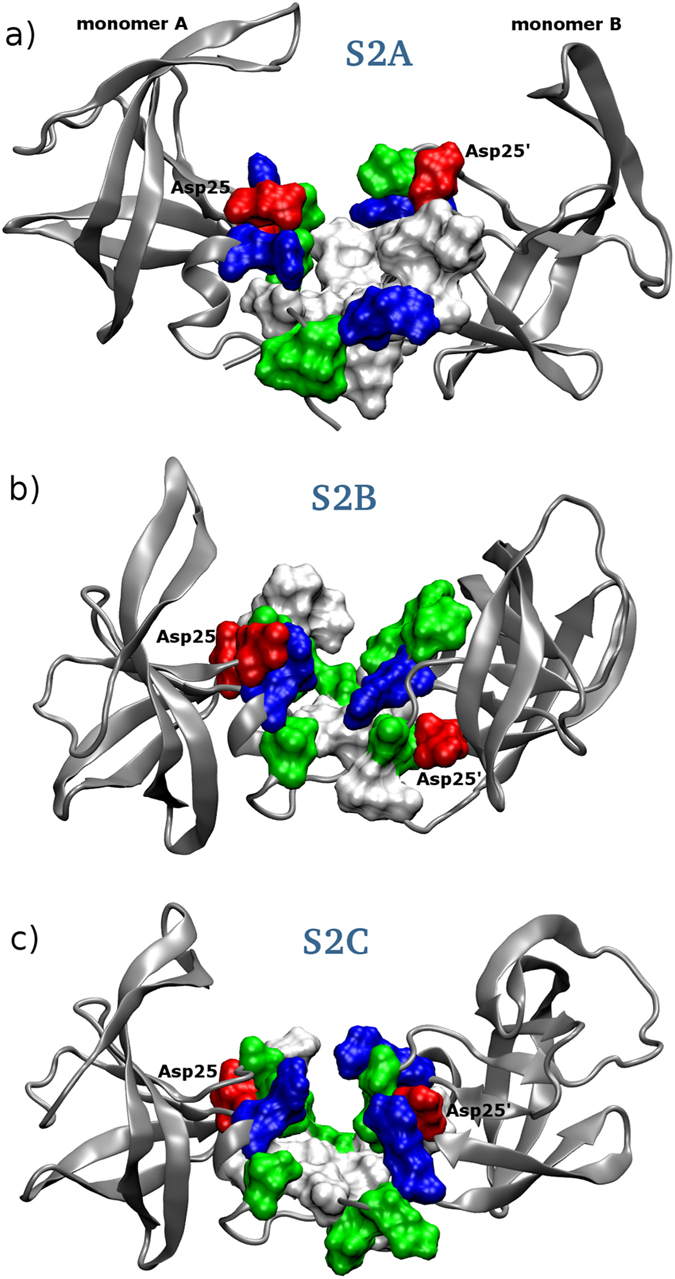
Three structures (panels a,b,c) representative of the ensemble of conformations in state S2, featuring the new cavity at the interface. The monomers A and B composing HIV1 protease are indicated as well as the two Asp from the catalytic triad. Hydrophobic residues are shown in white color, positively charged in blue, negatively charged in red and polar residues in green color. See text for details.

Free energy landscapes projected on collective variables monitoring the intermonomer distance, flap tips deformation, and RMSD from crystallographic interface are reported in Fig 2. The complex corresponds to the lowest minimum (state S1). With increasing intermonomer separation the system traverses a high free energy region, finding afterwards a second minimum (state S2) laying about 2.4 kcal/mol higher than S1. Remarkably, state S2 features a new cavity resembling a binding pocket (Fig. 3), that will be the focus of the analyses in the following sections.

Further increasing the intermonomer separation leads to a steep rise of the free energy, corresponding to the progressive loss of favorable interface contacts with the consequent penetration of more and more water molecules. The complete dissociation of the dimer, albeit occasionally observed in the simulations, is beyond the scope of this work due to the large computational cost required to obtain an acceptable sampling statistics and due to its possibly complex interplay with the (un)folding of each monomer^14^.

### The new open conformation is kinetically separated from the closed one

**Figure 4:**
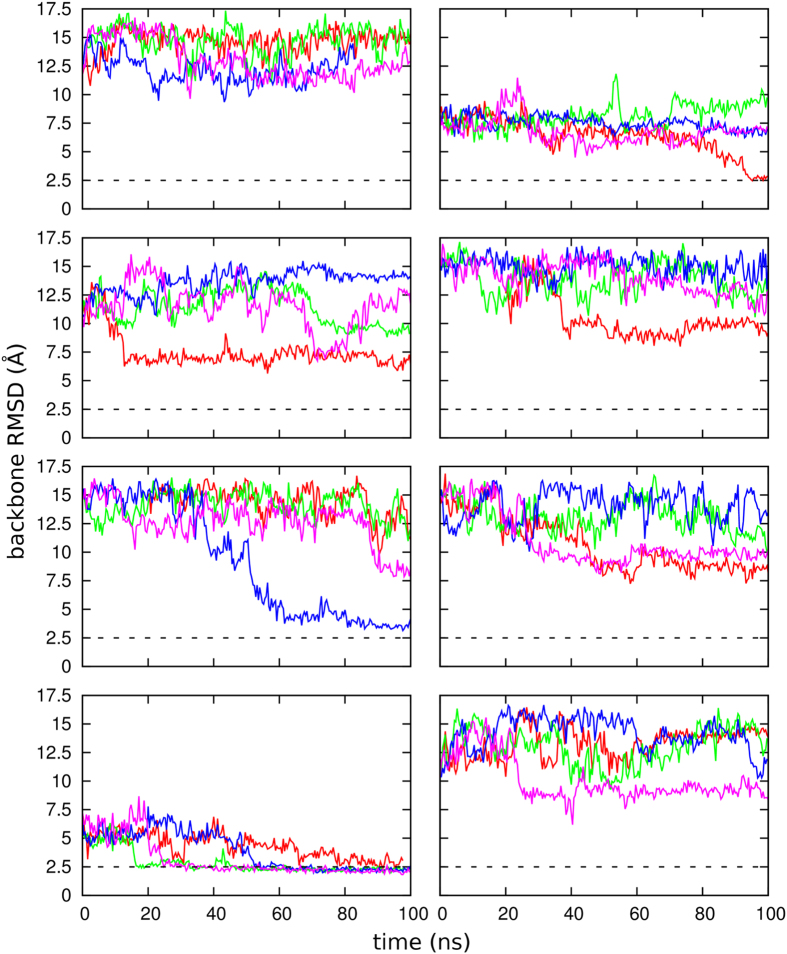
Evolution of the backbone RMSD with respect to the crystallographic complex during a total of 32 unbiased MD simulations started from 8 different structures (corresponding to the different panels) in basin S2.

To further investigate the stability of the new cavity beyond the results of enhanced sampling simulations, we performed 32 additional unbiased MD simulations starting from configurations of basin S2. 8 representative structures were extracted from state S2, with intermonomer distance between 3.2 and 3.8 nm and a backbone RMSD between 7 and 15 Å from the crystallographic complex. From each structure, 4 independent equilibrium simulations at 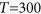 K were run for 100 ns. In 26 out of 32 cases, after 100 ns the dimer remained in conformation S2, with an open secondary cavity. In only 3 cases the dimer closed again to form a compact complex (with backbone RMSD <2.5 Å from the crystallographic structure), and in 3 more cases the dimer partially closed to a RMSD between 2.5 and 3 Å (Fig. 4). These results confirm the sizable local stability of configuration S2 and demonstrate the kinetic separation between free energy basins S1 and S2, consistently with the barrier revealed by enhanced sampling simulations (Fig. 2).

**Figure 5:**
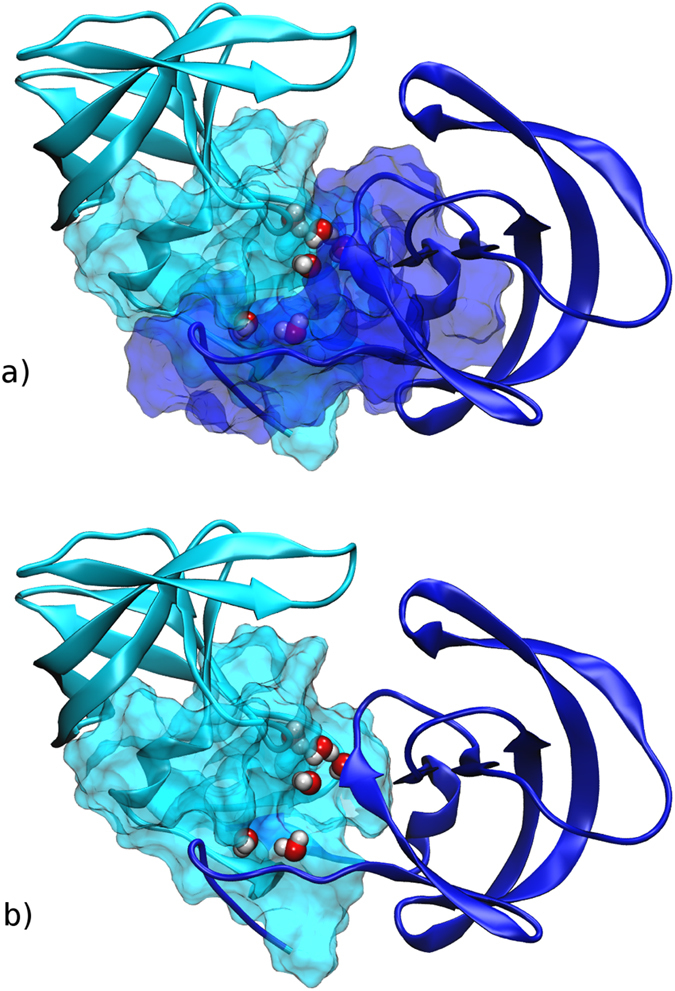
Example of dimer conformation in the transition region connecting the fully bound complex (state S1) with the partially open complex featuring the new binding pocket (state S2). Six water molecules are trapped at the dimer interface (dry in state S1), without contact with the bulk solvent. In (panel **a**) both monomers are represented in the same way, whereas in (panel **b**) the second one is shown only as a cartoon to allow a more direct view of water molecules.

Structures observed in the transition region between basins S1 and S2, laying at about 6 kcal/mol in the free energy landscape (Fig. 2, intermonomer distance 3.0–3.1 nm), feature the opening of a few channels at the interface close to the catylitic triads, large enough to accomodate about 3–6 water molecules (Fig. 5). In state S2, the new cavity contains instead a large number of water molecules (>20). Therefore, the closure of the dimer S2→S1 implies steps charaterized by the progressive expulsion of water, until reaching the structure of the crystallographic complex where the interface is dry. Note that even in simulations displaying closure of the dimer, the system remains trapped in a (metastable) configuration similar to the transition state described above, apart for a larger number of interfacial water molecules (5–10), their expulsion requiring a time scale longer than the simulated one of 100 ns. This confirms the preceeding analysis of the transition state: the expulsion of a few interfacial water molecules is a kinetic bottleneck for the transition S2→S1, the latter having a rate <10^6^ s^−1^. We remark that water molecules were also shown to play a key role in shaping the pathway and kinetics for peptide substrate binding to the catalytic cavity of HIV-1 protease^24^. In fact, water is increasingly acknowledged as central to biomolecular recognition and more general association processes^25^,^26^. Note that in ref. 27 the binding of the monomers was investigated by means of discrete MD simulations in implicit solvent, finding that several metastable conformations exist where the homodimer is imperfectly dimerized, however such conformations were characterized by disorder at the N- and C-terminal beta sheet whereas state S2 was not found, possibly due to the lack of an explicit treatement of the solvent.

### The transient pocket exists in an ensemble of conformations

Here we proceed to analyze in detail the structural features of metastable state S2, that displays the newly discovered transient cavity. Due to fluctuations during the molecular dynamics simulation, state S2 consists in an ensemble of conformations. For the sake of clarity we restrict the discussion to three representative geometries, labelled S2-A,B,C and represented in Fig. 3, that are the central structures of clusters spanning the conformational ensemble (see section Methods for details). The set of amino acids composing the pocket can vary depending on the geometry of the representative structure.

All three binding pockets have a hydrophobic bottom lined with polar and charged amino acids (Fig. 3a–c, respectively). The non-polar amino acids composing the bottom of binding pocket S2A are Leu5, Trp6, Gly27, Leu 97, Phe99, Leu5′, Pro9′, Val11′, Leu23′, Leu24′, Leu90′ and Ile93′. The walls of the pocket are mostly built by charged and polar amino acids as follows: Arg8, Arg 87, Arg8′, Arg87′, Asp25, Asp29, Asp25′, Thr26, Thr4′, Gln7′ and Thr26′. From the two catalytic aspartates only Asp25 is oriented towards the cavity and can interact with ligands.

The S2B binding pocket is narrower than S2A. From monomer A all hydrophobic residues appearing in S2A are still present, except Phe99. From monomer B there are Leu5′, Gly27′, Cys95′ and Leu97′. S2B has a smaller amount of charged residues - Asp29, Arg87 and Arg87′ – than S2A, but more polar ones: Thr4, Thr26, Thr91, Thr26′, Asn88′, Thr91′, Gln92′ and Thr96′. Thr96′ contributes rather hydrophobic contacts with its side chain methyl group pointing into the pocket, while its hydroxyl group is directed out of the cavity. As in the S2A only Asp25 is oriented towards the cavity and can interact with ligands (Fig. 3b).

The S2C binding pocket is the narrowest among all three binding sites and thereof potentially the most interesting for developing new HIV1 PR inhibitors (see also the next section). The bottom of S2C is composed of hydrophobic residues Ile3, Leu5, Gly27, Leu90, Leu97, Ile3′, Leu5′, Gly27′, Leu90′, Cys95′ and Leu97′. The hydrophobic bottom is surrounded by positively charged - Arg87, Arg8′ and Arg87′ - and polar residues - Thr4, Thr26, Thr91, Thr4′, Gln7′, Thr26′, Thr91′ and Thr96′. The side chains of both catalytic aspartates are pointing out of the cavity.

Compared to S1, structures belonging to metastable state S2 are characterized not only by the opening of the new cavity, but also by the partial rotation of each monomer around an axis passing through its center of mass and perpendicular to the plane of the N- and C-terminal beta sheet (see Fig. 3). On the other hand, the structure of each monomer in structures S2A/B/C remains very similar to state S1: the backbone RMSD is at most 3.3 Å, with the deviation stemming mainly from the flap (residues 41 to 59 have RMSD up to 1.9 Å) and from terminal residues involved in the formation of the interdigitated beta sheet (residues 1 to 9 and 91 to 99 have RMSD of 2.2–3.5 Å), since the latter bends as a hinge in S2, whereas the remaining bulk of the monomer deviates by 1.1–1.5 Å only. Therefore state S2 can be seen as a deformation of S1 where each monomer behaves as a rather rigid unit and does not unfold. Our results also confirm that the N- and C-terminal beta sheet is the interface region most difficult to break down. We remark that the scenario is consistent with previous experimental^28^ and computational^29^,^30^ works that suggested the existence of a relatively stable intermediate folded monomer (with flexible terminal residues) on the pathway leading from the unfolded monomers to the folded dimer.

### Druggability, docking of inhibitors and their binding free energy

We now approach the question of how likely is the design of small-molecules able to bind tightly to the new pocket. As discussed in the preceeding section, the size and amino acid composition of the cavity appear favorable in this respect, and more quantitative analysis in terms of druggability scores brings further support to this claim. We applied to structures S2A/B/C two different and widespread algorithms, fpocket^31^ and dogsitescorer^32^: both employ the size, shape, and chemical features of putative binding pockets (automatically identified from the structure of the protein) to score their druggability, and they have been validated on extensive data sets including hundreds of pharmaceutical targets^32^,^33^. fpocket gives druggability scores of 0.51, 0.59 and 0.89 for structures S2A/B/C, respectively, to be compared with an average score of 0.65 (standard deviation 0.14) over 24 HIV-1 protease holo structures of the catalytic cavity and with a score of 0.44 for the corresponding apo structure^33^. dogsitescorer provides scores of 0.83, 0.78, and 0.75 for structures S2A/B/C, respectively, to be compared with scores between 0.6 and 0.7 for the catalytic cavity^32^. Therefore the two algorithms agree in predicting a very likely druggability for the newly discovered binding pocket.

**Figure 6:**
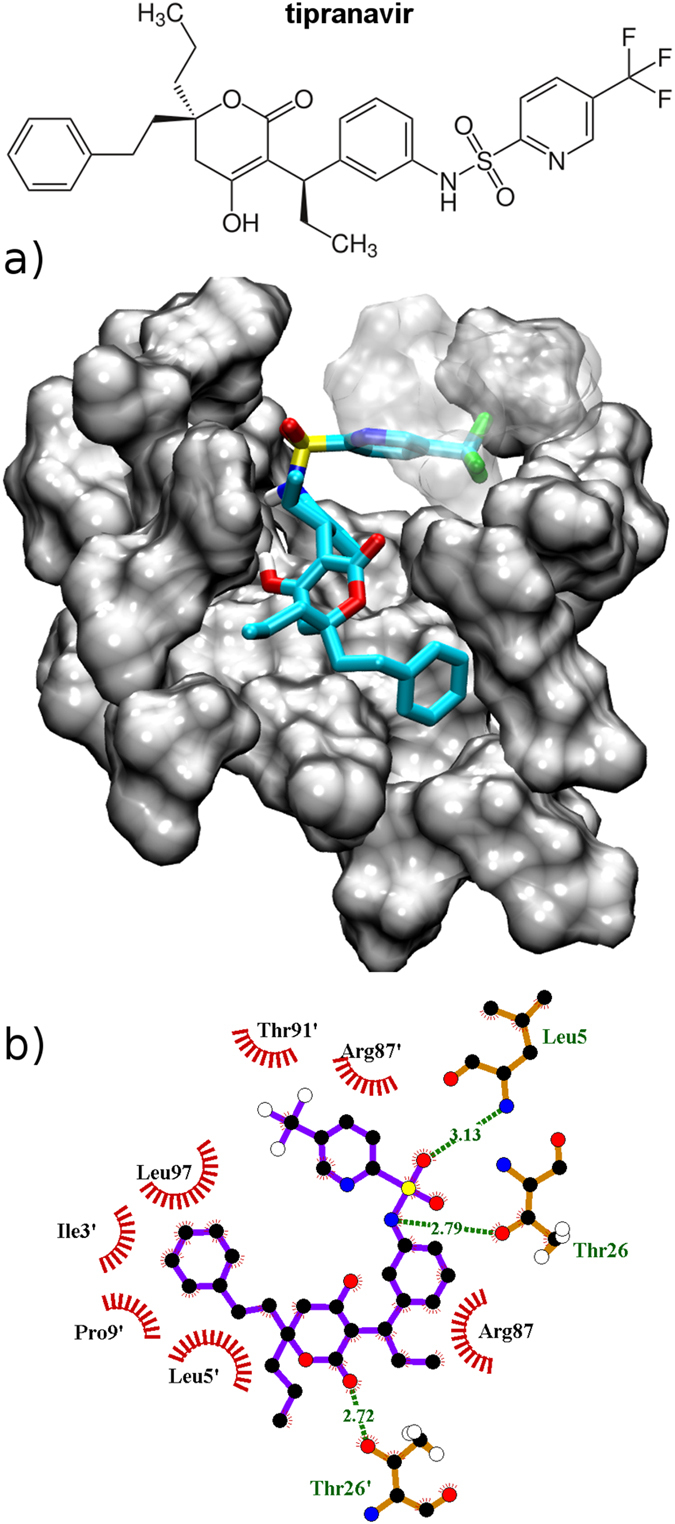
(**a**) Binding of tipranavir to the new pocket in conformation S2C. For the sake of clarity some protease residues are shown as transparent. The color code is as follows: C atoms - cyan, N atoms - blue, O atoms - red, F atoms - green and S atoms - yellow. (**b**) Tipranavir interactions with the binding site represented employing the software Ligplot+^60^.

**Figure 7:**
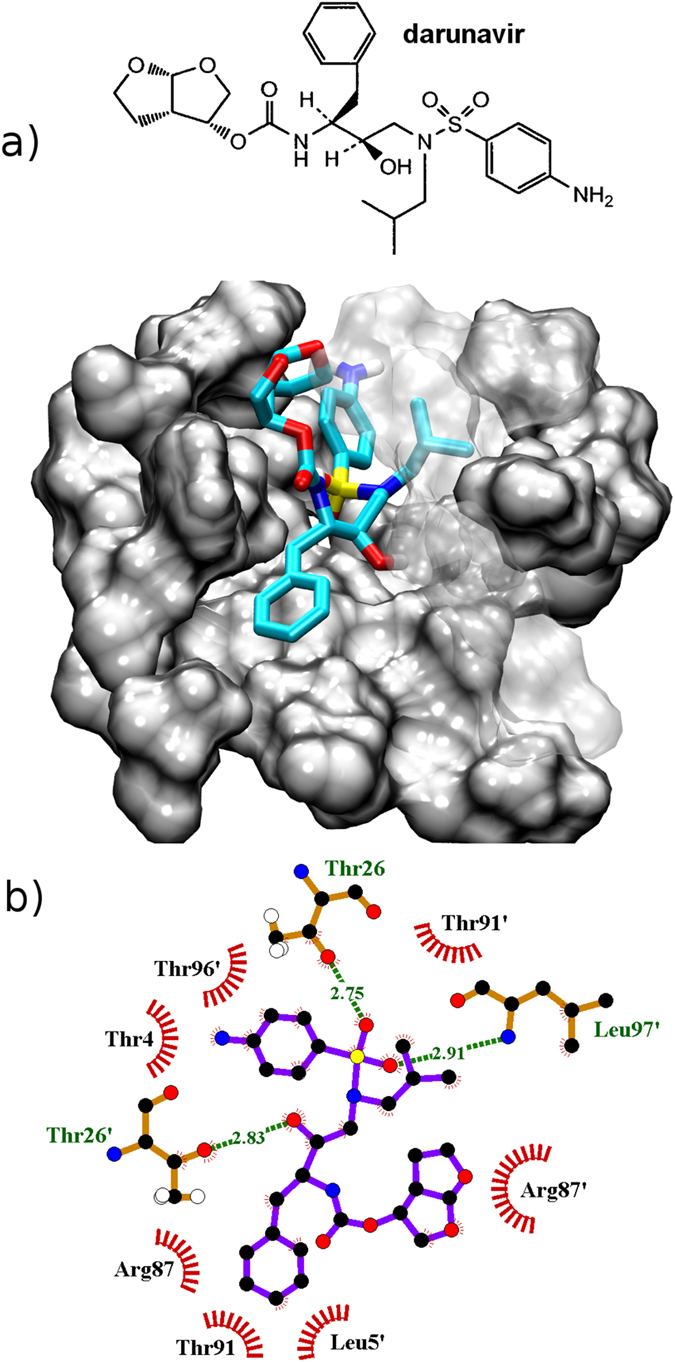
(**a**) Binding of darunavir to the new pocket in conformation S2C. (**b**) Darunavir interactions with the binding site represented employing the software Ligplot+^60^.

To gain further insight into the possibility of designing dimerization inhibitors, we performed some preliminary docking calculations: here, without any attempt to be exhaustive, we ask the question of how drug molecules can accomodate themselves in the new pocket, and what favorable interatomic contacts can they establish. Out of a number of FDA approved drugs, we decided to dock tipranavir (Fig. 6) and darunavir (Fig. 7) as they were reported to inhibit also protease dimerization in addition to enzymatic activity^16^. We employed the program Autodock Vina^34^ (see Methods section). We docked both inhibitors to each of the three binding pockets - S2 A, B and C. The binding affinities obtained by docking were −5.8 kcal/mol, −7.3 kcal/mol and −7.8 kcal/mol for darunavir, respectively. Tipranavir shows higher binding affinities than darunavir: −7.8 kcal/mol, −7.9 kcal/mol and −9.2 kcal/mol, respectively.

For both inhibitors structure S2C displays the highest docking affinity: starting from the best docking pose we proceeded to estimate more accurately the binding free energy to the latter conformation of the partially open dimer, comparing it with that for the catalytic cavity of the closed dimer. To this aim we employed the Molecular Mechanics - Generalized Born Surface Area (MM-GBSA) method^35^,^36^, that proved able to rank correctly the binding affinity of the series of FDA-approved HIV-1 protease inhibitors^37^. The details of the procedure are reported as Supporting Information. As shown in Table 1, the drugs bind less strongly in the new cavity compared to the catalytic pocket, however the difference is relatively small: 3 kcal/mol for tipranavir and only 1 kcal/mol for darunavir, in all cases within the statistical uncertainty of the calculations. Thus it is fair to state that affinity for the new cavity is comparable to that for the crystallographic pocket, a remarkable result considering that the drugs were designed to aim at the latter. From Table 1 it is clear that binding to the new cavity entails a smaller penalty in configurational entropy, as estimated through normal mode analysis (see Supporting Information for details and a decomposition into the contribution of different protease residues). To summarize, our calculations suggest that tipranavir and darunavir might bind to the new cavity in addition to the catalytic one, albeit with a possibly much lower population. Due to the imperfect accuracy of state-of-the-art force fields and to the error bar of free energy calculations, it is arduous to estimate the precise relative populations of the two binding modes. As a consequence, it is not our intention to claim a compelling identification of the structural basis of the dimerization inhibition effect experimentally observed for these drugs: to this aim, future experimental proofs will be mandatory. On the other hand, our results clearly show that the new cavity could be able to bind drug-like molecules, therefore it is worth investing research efforts in the design of new dimerization inhibitors specifically targeting with high affinity the mis-bound dimer, thus capturing the inactive protease conformation.

We report here detailed binding interactions between each inhibitor and the S2C binding pocket, as observed during the MD simulations employed in the MM-GBSA calculation (only few contacts differ from the starting configuration obtained with Autodock Vina). Tipranavir is a sulfonamide-containing dyhydropyrone and a nonpeptidic protease inhibitor^38^. In our simulation the phenylethyl moiety of tipranavir forms hydrophobic interactions with Leu97, Ile3′ and Pro9′ (see Fig. 6b). The propyl group is pointing out to the solvent and thus makes no hydrophobic interactions. The 5-hydroxy-4H pyrone moiety makes hydrophobic interactions with Leu5′, and its oxo group forms a hydrogen bond with the Thr26′ hydroxyl group. The ethyl moiety is pointing out of the cavity, however it forms hydrophobic contact with the Arg87 C*δ* atom. The benzyl group sits in the hydrophobic pocket formed by Leu97 and by Thr26 and Thr26′ methyl groups. The amino part from the sulfonamide moiety makes H-bonds with Thr26 hydroxyl group, while one of the oxygens forms H-bonds with the Leu5 backbone amino group. The pyridine group forms hydrophobic interactions with a methyl group of Thr91′ and main chain CH_2_ groups of Arg87′. The 5-trifluoromethyl-2-sulfoxy-pyridine moiety has a negative partial charge and it is located next to a positively charged Arg87′.

Darunavir is a bis-tetrahydrofuranylurethane-containing nonpeptidic protease inhibitor^39^. Our simulation shows that aniline in darunavir makes hydrophobic interactions with the C*α* and C*β* atoms of Thr4 and with the methyl group of Thr96′ (see Fig. 7b). The aniline amino group is pointing out to the water solution and has no contacts with the residues of S2C binding pocket. One of the sulfonamide group oxygens forms a H-bond with Leu97′ backbone nitrogen, while the other makes a H-bond with Thr26 hydroxyl group. The methylisopropyl group forms hydrophobic interactions with the Arg87′ C*β*, C*γ*, C*δ* atoms and with Thr9′ methyl group. The hydroxyl group of darunavir makes a H-bond with the hydroxyl group of Thr26′. The benzyl group sits in the hydrophobic pocket formed by Leu5′, Leu97′, C*γ* of Arg87 and the methyl groups of Thr26 and Thr91. The carbamate moiety and the bis tetrahydrofuran group are exposed to the solvent and have no close interactions with the studied protein.

It is important to remark that molecules binding to the newly discovered pocket, thus capturing the partially formed dimer into the functionally inactive state, could be overlooked as dimerization inhibitors by techniques like fluorescence. This is because the dimer is not completely destroyed, in particular the terminal beta sheet remains intact.

## Discussion

**Figure 8:**
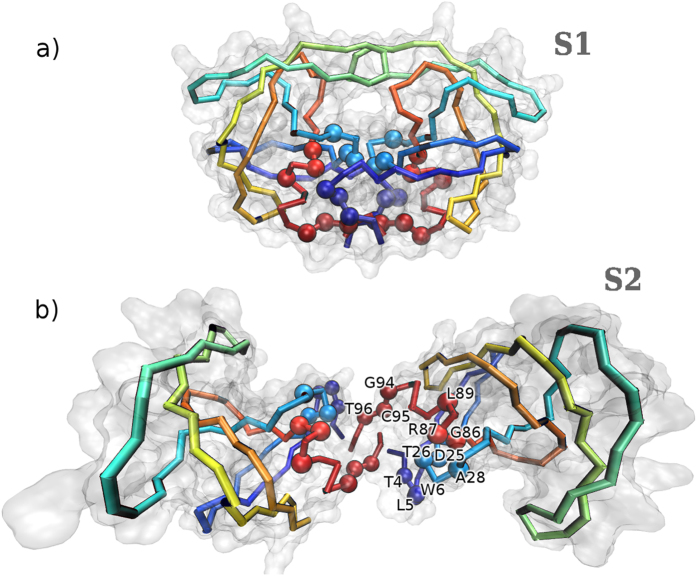
Protease residues (spheres) undergoing sizable chemical exchange on the *μ*s-ms time scale in ^1^H and ^15^N nuclear spin transverse relaxation measurements in ref. 9, in (a) the crystallographic protease structure, and (b) the partially open structure S2C.

The present study provides atomically detailed insight on the binding mechanism between protease monomers, and it may help explaining several remarkable experimental results that so far are not fully understood. Previous ^1^H and ^15^N nuclear spin transverse relaxation measurements revealed, somehow unexpectedly, that a series of protease residues display sizable chemical exchange on the *μ*s-ms time scale, pointing to slow (activated) conformational changes^9^. Besides flap tips residues, whose flexibility is expected, the set includes residues 4–6, 25, 26, 28, 86, 87, 89, 94–96: as displayed in Fig. 8, all of the latter amino acids belong to the surface of the newly identified secondary cavity, therefore they undergo a drastic change of chemical environment with respect to the crystallographic structure upon the partial opening of the dimer. Other residues displaying slow chemical exchange include 32-35, 76, 77, 82, and 84: they are also affected by the opening transition, in this case due to the flaps moving to rest upon the bulk of their respective monomer. Further measurements on ^13^C relaxation point to slow conformational changes at residues 3, 5, and 97, again lining the new cavity^10^. In a more recent study addressing the autoprocessing of the enzyme (albeit featuring a four-residue N-terminal extension), paramagnetic relaxation enhancement measurements could not be interpreted based solely on thermal fluctuations of the mature dimer: as suggested by a simple modelling of the complex as formed by two rigid monomers, the latter could partially rotate with respect to each other giving rise to sizably populated misbound dimers^40^. Here we argue that the opening of the new pocket in configuration S2 could be related to such transient conformations.

Overall, our findings are thus compatible with experimental measurements and may help rationalizing them. Our results also confirm that in the mature enzyme the strength of intermonomer interactions grow passing from the flap tips to the complex core to the terminal beta sheet. From a mechanical point of view, it appears clear that this hierarchy *must* lead to relatively easy transient opening events of the flap tips, a well-known phenomenon, followed by less frequent transient opening of the new cavity S2, followed by yet more harduous separation (and unfolding) of the terminal beta sheet. In other words, it could be expected beforehand that (overcoming a free energy barrier) the dimer would be able to open with the monomers pivoting around the hinge formed by the terminal beta sheet. This very observation leads us to suggest that other protein complexes featuring a similar hierarchy in interface interactions could display the transient exposure of new binding pockets, as in the case studied here, what could possibly result in new pharmaceutical targets.

By exploiting a combination of enhanced sampling molecular dynamics, druggability scores, molecular docking, and MM-GBSA free energy calculations, we suggest that state S2 could be an interesting new pharmaceutical target. The compelling identification of potent dimerization inhibitors targeting the new site, even just in silico, requires more extensive work that is beyond the scope of the present article. To this aim, we provide the scientific community with the ensemble of structures corresponding to the new binding pocket (downloadable as Supporting Information), in the hope of triggering the design of a new class of drug molecules. So far, structure-assisted drug design has been crucially important for developing inhibitors of HIV-1 protease^1^,^2^. We hope that the new structural insight we provide here will help to renew research efforts, leading to improved antiviral treatments.

## Methods

### Molecular dynamics

Molecular dynamics simulations explicitly included all protein atoms, described with a recent accurate forcefield^41^, as well as 5095 water molecules^42^ (for a total of 78,400 atoms in a periodically repeated cubic box of side 9.3 nm), employing the software gromacs^43^. One of the residues in the Asp25/25′ catalytic dyad was protonated, according to existing evidences for the free enzyme^44^,^45^,^46^. Five chlorine ions were added to enforce charge neutrality. We started from the crystallographic structure 1AID^23^, employing a timestep of 2 fs and constraining all covalent bonds. After equilibrating for 1 ns at *T* = 300 K and *p* = 1 atm employing the stochastic velocity rescaling thermostat^47^ and Berendsen’s barostat^48^, the deviation of the final structure from the initial one is only 1.7 Å C_*α*_ RMSD. The following simulations were performed in the *NVT* ensemble.

### Bias-exchange metadynamics

We enhanced the sampling of the slow dissociation/association events between protease monomers exploiting bias-exchange metadynamics^21^, as implemented in the plugin Plumed^49^. We employed the following five collective variables (where the reference structure for RMSD calculations is the geometry of the complex after 1 ns of equilibration):

CV1 = distance between centers of mass of monomers (excluding flaps and the terminal beta sheet: C*α* atoms of residues 10–40 and 60–90 of both monomers);

CV2 = RMSD of hydrophobic side chain carbon atoms at the interface (Leu5,24,90,97 and Thr26 of both monomers);

CV3 = RMSD of catalyitic triads (backbone and C*β* atoms of Asp25, Thr26, Gly27 of both monomers);

CV4 = RMSD of flap tips (backbone and C*β* atoms of residues 49–52 of both monomers);

CV5 = RMSD of the terminal beta sheet (backbone and C*β* atoms of residues 1–4 and 96–99 of both monomers).

We employed four replicas, three of them biasing one CV each among CV1, CV3, CV4, and the fourth replica left unbiased. A first simulation was performed without any restriction over the values of the CVs, and allowed to explore the overall conformational landscape for monomer-monomer interaction, including events where the dimer fully dissociated. Next, we performed a second simulation constraining the value of CV5 to be smaller than 2 Å, in order to avoid the complete dissociation and to focus the sampling on the regions of the landscape including states S1 and S2. Note that the latter threshold is never surpassed during long unconstrained equilibrium MD simulations started from state S2 (see below).

The 4-dimensional free energy landscape as a function of CVs 1–4 was reconstructed using the approach in ref. 22, based on the weighted histogram analysis method, as implemented in the software Metagui^50^. Parallel growth of each bias profile is observed after 28 ns of simulation per replica, therefore the trajectory data between 28 and 68 ns is employed for reconstructing the landscape. 150 structural clusters were obtained by the k-medoids algorithm, where each cluster is visited at least 10 times during the simulation (with trajectories being recorded every 5 ps). The robustness of the results were checked by changing the number of clusters between 100 and 200, obtaining very similar results, as well as by comparing the free energy difference between states S1 and S2 computed by WHAM with that estimated by the relative populations in the unbiased replica, obtaining a deviation smaller than 0.7 kcal/mol. For further analyses and docking, the ensemble of structures forming state S2 was partitioned in clusters (based on a backbone RMSD cutoff of 4 Å) and the three resulting cluster centers, labeled S2A,B,C, were considered.

### Docking

Molecular docking was performed using the software AutoDock Vina^34^. Ligands had fully flexible torsion degrees of freedom, while the receptor side chains were inflexible. Protein and ligand input files were prepared by AutoDockTools^51^. The initial 3D structure of darunavir was extracted from the PDB file 3S53^52^, whereas tipranavir was taken out of the PDB file 3SPK^53^. Non-polar hydrogen atoms of the protein and the ligands were merged. The center of grid was placed at the X = −2.036 Å, Y = −1.903 Å and Z = 0.031 Å (with respect to the geometry provided as supporting information). Grid dimensions were 20 Å × 18 Å × 20 Å and the spacing between the grid points was set to 1 Å. The exhaustiveness parameter of the global search was set at 8. Twenty binding modes were generated for each inhibitor and each binding site.

### MM-GBSA binding free energy calculations

The binding free energy of tipranavir and darunavir to either the crystallographic catalytic pocket or the new binding pocket was estimated using the MM-GBSA technique^35^,^54^,^55^,^56^ employing the AMBER14 code^57^. To this aim, we performed MD simulations of the solvated complex including about 70,000 atoms employing the parm14SB force field for the protein^58^, TIP3P^42^ for the water and Joung & Cheatham parameters for ions^59^. For each complex we performed a sequence of simulations: structural relaxation with soft restraints, followed by annealing up to 340 K in 2 ns, followed by 5 ns of partially restrained MD at 310 K. The protocol is the same as in ref. 54 and is described in details in Supporting Information, together with the formulas employed to estimate the binding free energy.

## Additional Information

**How to cite this article**: Pietrucci, F. *et al.* HIV-1 Protease Dimerization Dynamics Reveals a Transient Druggable Binding Pocket at the Interface. *Sci. Rep.* **5**, 18555; doi: 10.1038/srep18555 (2015).

## Supplementary Material

Supplementary Information

Supporting Information

## Figures and Tables

**Figure 1:**
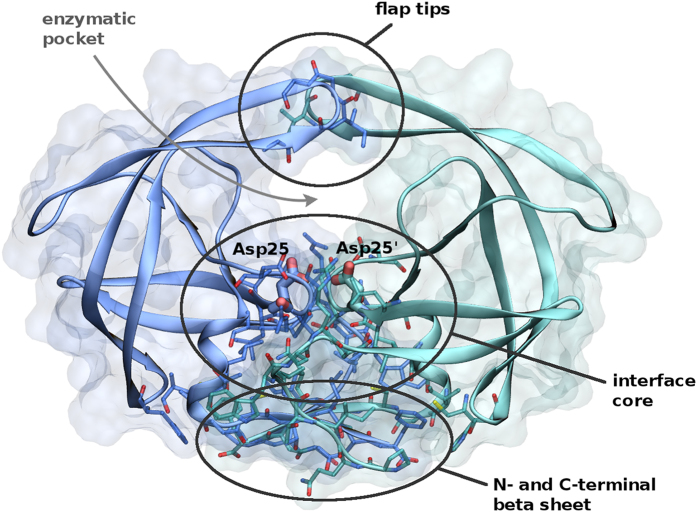
Crystallographic structure of the HIV-1 protease dimeric complex (pdb entry 1AID^23^). The enzymatic cavity and the three main regions forming the interface are indicated.

**Table 1:** 

	opt			MD		
complex	Δ*G*_solv_	*T*Δ*S*_conf_	Δ*G*_b_	Δ*G*_solv_	*T*Δ*S*_conf_	Δ*G*_b_
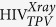	−63.0	−31.1	−31.9	−56.5 (0.6)	−33.2 (2.5)	−23.3 (3.1)
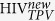	−51.7	−21.9	−29.8	−46.2 (1.0)	−26.0 (2.1)	−20.2 (3.1)
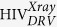	−51.8	−34.4	−17.4	−45.2 (0.7)	−33.2 (1.2)	−12.0 (1.9)
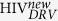	−43.9	−21.2	−22.7	−33.2 (1.0)	−22.5 (1.4)	−10.7 (2.4)

Binding free energy (Δ*G*_b_, kcal/mol) from MM-GBSA for HIV-1 protease in complex with tipranavir (TPV) and darunavir (DRV), either bound to the catalytic pocket of the X-ray crystallographic structure or bound to the new binding pocket of the partially open dimer (structure S2C). The free energy is computed starting from the best docking pose (see text), and employing either the geometry after structural relaxation in presence of soft restraints or geometries extracted from MD simulations of 5 ns duration each. See Supporting Information for the decomposition into solvation free energy (Δ*G*_solv_) and configurational entropy (*T*Δ*G*_conf_) and for per-residue contributions.

## References

[b1] Wlodawer A. & Vondrasek J. Inhibitors of hiv-1 protease: A major success of structure-assisted drug design 1. Annu. Rev. Bioph. Biom. 27, 249–284 (1998). 10.1146/annurev.biophys.27.1.249 9646869

[b2] Pokorná J., Machala L., Řezáčová P. & Konvalinka J. Current and novel inhibitors of hiv protease. Viruses 1, 1209–1239 (2009). 21994591 10.3390/v1031209 PMC3185513

[b3] Jubb H., Higueruelo A. P., Winter A. & Blundell T. L. Structural biology and drug discovery for protein-protein interactions. Trends Pharmacol. Sci. 33, 241–248 (2012). 22503442 10.1016/j.tips.2012.03.006

[b4] Durrant J. D. & McCammon J. A. Molecular dynamics simulations and drug discovery. BMC Biol. 9, 71 (2011). 22035460 10.1186/1741-7007-9-71 PMC3203851

[b5] Bowman G. R. & Geissler P. L. Equilibrium fluctuations of a single folded protein reveal a multitude of potential cryptic allosteric sites. Proc. Natl. Acad. Sci. USA 109, 11681–11686 (2012). 22753506 10.1073/pnas.1209309109 PMC3406870

[b6] Schames J. R. *et al.* Discovery of a novel binding trench in hiv integrase. J. Med. Chem. 47, 1879–1881 (2004). 15055986 10.1021/jm0341913

[b7] Summa V. *et al.* Discovery of raltegravir, a potent, selective orally bioavailable hiv-integrase inhibitor for the treatment of hiv-aids infection. J. Med. Chem. 51, 5843–5855 (2008). 18763751 10.1021/jm800245z

[b8] Wassman C. D. *et al.* Computational identification of a transiently open l1/s3 pocket for reactivation of mutant p53. Nat. Commun. 4, 1407 (2013). 23360998 10.1038/ncomms2361 PMC3562459

[b9] Ishima R., Freedberg D. I., Wang Y.-X., Louis J. M. & Torchia D. A. Flap opening and dimer-interface flexibility in the free and inhibitor-bound hiv protease, and their implications for function. Structure 7, 1047–S12 (1999). 10508781 10.1016/s0969-2126(99)80172-5

[b10] Ishima R., Louis J. M. & Torchia D. A. Characterization of two hydrophobic methyl clusters in hiv-1 protease by nmr spin relaxation in solution. J. Mol. Biol. 305, 515–521 (2001). 11152609 10.1006/jmbi.2000.4321

[b11] Todd M. J., Semo N. & Freire E. The structural stability of the hiv-1 protease. J. Mol. Biol. 283, 475–488 (1998). 9769219 10.1006/jmbi.1998.2090

[b12] Hayashi H. *et al.* Dimerization of hiv-1 protease occurs through two steps relating to the mechanism of protease dimerization inhibition by darunavir. Proc. Natl. Acad. Sci. USA 111, 12234–12239 (2014). 25092296 10.1073/pnas.1400027111 PMC4142999

[b13] Camarasa M.-J., Velázquez S., San-Félix A., Pérez-Pérez M.-J. & Gago F. Dimerization inhibitors of hiv-1 reverse transcriptase, protease and integrase: a single mode of inhibition for the three hiv enzymes? Antivir. Res. 71, 260–267 (2006). 16872687 10.1016/j.antiviral.2006.05.021

[b14] Cardinale D. *et al.* Homodimeric enzymes as drug targets. Curr. Med. Chem. 17, 826–846 (2010). 20156173 10.2174/092986710790712156

[b15] Ershov P. *et al.* Kinetic and thermodynamic analysis of dimerization inhibitors binding to hiv protease monomers by surface plasmon resonance. Biochemistry-Moscow 6, 94–97 (2012). 10.18097/pbmc20125801043 22642151

[b16] Koh Y. *et al.* Potent inhibition of hiv-1 replication by novel non-peptidyl small molecule inhibitors of protease dimerization. J. Biol. Chem. 282, 28709–28720 (2007). 17635930 10.1074/jbc.M703938200

[b17] Koh Y. *et al.* *In vitro* selection of highly darunavir-resistant and replication-competent hiv-1 variants by using a mixture of clinical hiv-1 isolates resistant to multiple conventional protease inhibitors. J. Virol. 84, 11961–11969 (2010). 20810732 10.1128/JVI.00967-10 PMC2977898

[b18] Aoki M. *et al.* Loss of the protease dimerization inhibition activity of tipranavir (tpv) and its association with the acquisition of resistance to tpv by hiv-1. J. Virol. 86, 13384–13396 (2012). 23015723 10.1128/JVI.07234-11 PMC3503118

[b19] Huang D. & Caflisch A. How does darunavir prevent hiv-1 protease dimerization? J. Chem. Theory Comput. 8, 1786–1794 (2012). 26593669 10.1021/ct300032r

[b20] Zhang Y. *et al.* Structures of darunavir-resistant hiv-1 protease mutant reveal atypical binding of darunavir to wide open flaps. ACS Chem. Biol. 9, 1351–1358 (2014). 24738918 10.1021/cb4008875 PMC4076034

[b21] Piana S. & Laio A. A bias-exchange approach to protein folding. J. Phys. Chem. B 111, 4553–4559 (2007). 17419610 10.1021/jp067873l

[b22] Marinelli F., Pietrucci F., Laio A. & Piana S. A kinetic model of trp-cage folding from multiple biased molecular dynamics simulations. PLoS Comput. Biol. 5, e1000452 (2009). 19662155 10.1371/journal.pcbi.1000452 PMC2711228

[b23] Rutenber E. *et al.* Structure of a non-peptide inhibitor complexed with hiv-1 protease. developing a cycle of structure-based drug design. J. Biol. Chem. 268, 15343–6 (1993). 8340363

[b24] Pietrucci F., Marinelli F., Carloni P. & Laio A. Substrate binding mechanism of hiv-1 protease from explicit-solvent atomistic simulations. J. Am. Chem. Soc. 131, 11811–11818 (2009). 19645490 10.1021/ja903045y

[b25] Raschke T. M. Water structure and interactions with protein surfaces. Curr. Opin. Struct. Biol. 16, 152–159 (2006). 16546375 10.1016/j.sbi.2006.03.002

[b26] Berne B. J., Weeks J. D. & Zhou R. Dewetting and hydrophobic interaction in physical and biological systems. Annu. Rev. Phys. Chem. 60, 85 (2009). 18928403 10.1146/annurev.physchem.58.032806.104445 PMC3898792

[b27] Kimura S., Caldarini M., Broglia R. A., Dokholyan N. V. & Tiana G. The maturation of hiv-1 protease precursor studied by discrete molecular dynamics. Proteins 82, 633–639 (2014). 24123234 10.1002/prot.24440 PMC4113227

[b28] Noel A. F. *et al.* The folding free-energy surface of hiv-1 protease: insights into the thermodynamic basis for resistance to inhibitors. J. Mol. Biol. 387, 1002–1016 (2009). 19150359 10.1016/j.jmb.2008.12.061 PMC2756696

[b29] Levy Y. & Caflisch A. Flexibility of monomeric and dimeric hiv-1 protease. J. Phys. Chem. B 107, 3068–3079 (2003).

[b30] Bonomi M., Barducci A., Gervasio F. L. & Parrinello M. Multiple routes and milestones in the folding of hiv-1 protease monomer. PloS one 5, e13208 (2010). 20967249 10.1371/journal.pone.0013208 PMC2954147

[b31] Le Guilloux V., Schmidtke P. & Tuffery P. Fpocket: an open source platform for ligand pocket detection. BMC Bioinformatics 10, 168 (2009). 19486540 10.1186/1471-2105-10-168 PMC2700099

[b32] Volkamer A., Kuhn D., Grombacher T., Rippmann F. & Rarey M. Combining global and local measures for structure-based druggability predictions. J. Chem. Inf. Model. 52, 360–372 (2012). 22148551 10.1021/ci200454v

[b33] Schmidtke P. & Barril X. Understanding and predicting druggability. a high-throughput method for detection of drug binding sites. J. Med. Chem. 53, 5858–5867 (2010). 20684613 10.1021/jm100574m

[b34] Trott O. & Olson A. J. Autodock vina: improving the speed and accuracy of docking with a new scoring function, efficient optimization, and multithreading. J. Comput. Chem. 31, 455–461 (2010). 19499576 10.1002/jcc.21334 PMC3041641

[b35] Kollman P. A. *et al.* Calculating structures and free energies of complex molecules: Combining molecular mechanics and continuum models. Acc. Chem. Res. 33, 889–897 (2000). 11123888 10.1021/ar000033j

[b36] Srinivasan J., Cheatham T. E. III, Cieplak P., Kollman P. A. & Case D. A. Continuum Solvent Studies of the Stability of DNA, RNA, and Phosphoramidate-DNA Helices. J. Am. Chem. Soc. 120, 9401–9409 (1998).

[b37] Wright D. W., Hall B. A., Kenway O. A., Jha S. & Coveney P. V. Computing clinically relevant binding free energies of hiv-1 protease inhibitors. J. Chem. Theory Comput. 10, 1228–1241 (2014). 24683369 10.1021/ct4007037 PMC3966525

[b38] Poppe S. *et al.* Antiviral activity of the dihydropyrone pnu-140690, a new nonpeptidic human immunodeficiency virus protease inhibitor. Antimicrob. Agents Ch. 41, 1058–1063 (1997). 10.1128/aac.41.5.1058 PMC163850 9145869

[b39] Koh Y. *et al.* Novel bis-tetrahydrofuranylurethane-containing nonpeptidic protease inhibitor (pi) uic-94017 (tmc114) with potent activity against multi-pi-resistant human immunodeficiency virus *in vitro*. Antimicrob. Agents Ch. 47, 3123–3129 (2003). 10.1128/AAC.47.10.3123-3129.2003 PMC201142 14506019

[b40] Tang C., Louis J. M., Aniana A., Suh J.-Y. & Clore G. M. Visualizing transient events in amino-terminal autoprocessing of hiv-1 protease. Nature 455, 693–696 (2008). 18833280 10.1038/nature07342 PMC2798589

[b41] Lindorff-Larsen K. *et al.* Improved side-chain torsion potentials for the amber ff99sb protein force field. Proteins 78, 1950–1958 (2010). 20408171 10.1002/prot.22711 PMC2970904

[b42] Jorgensen W. L., Chandrasekhar J., Madura J. D., Impey R. W. & Klein M. L. Comparison of simple potential functions for simulating liquid water. J. Chem. Phys. 79, 926–935 (1983).

[b43] Hess B., Kutzner C., van der Spoel D. & Lindahl E. Gromacs 4: Algorithms for highly efficient, load-balanced, and scalable molecular simulation. J. Chem. Theory Comput. 4, 435–447 (2008). 26620784 10.1021/ct700301q

[b44] Piana S. & Carloni P. Conformational flexibility of the catalytic asp dyad in hiv-1 protease: An ab initio study on the free enzyme. Proteins 39, 26–36 (2000). 10737924 10.1002/(sici)1097-0134(20000401)39:1<26::aid-prot3>3.0.co;2-n

[b45] Carnevale V., Raugei S., Piana S. & Carloni P. On the nature of the reaction intermediate in the hiv-1 protease: a quantum chemical study. Comput. Phys. Commun. 179, 120–123 (2008).

[b46] McGee T. D., Edwards J. & Roitberg A. E. ph-remd simulations indicate that the catalytic aspartates of hiv-1 protease exist primarily in a monoprotonated state. J. Phys. Chem. B 118, 12577–12585 (2014). 25340507 10.1021/jp504011c

[b47] Bussi G., Donadio D. & Parrinello M. Canonical sampling through velocity rescaling. J. Chem. Phys. 126, 014101 (2007). 17212484 10.1063/1.2408420

[b48] Berendsen H. J. C., Postma J. P. M., van Gunsteren W. F., DiNola A. & Haak J. R. Molecular dynamics with coupling to an external bath. J.Chem. Phys. 81, 3684–3690 (1984).

[b49] Bonomi M. *et al.* Plumed: a portable plugin for free energy calculations with molecular dynamics. Comput. Phys. Commun. 180, 1961–1972 (2009).

[b50] Biarnés X., Pietrucci F., Marinelli F. & Laio A. Metagui. a vmd interface for analyzing metadynamics and molecular dynamics simulations. Comput. Phys. Commun. 183, 203–211 (2012).

[b51] Seeliger D. & de Groot B. L. Ligand docking and binding site analysis with pymol and autodock/vina. J. Comput. Aid. Mol. Des. 24, 417–422 (2010). 10.1007/s10822-010-9352-6 PMC2881210 20401516

[b52] Tie Y. *et al.* Critical differences in hiv-1 and hiv-2 protease specificity for clinical inhibitors. Protein Sci. 21, 339–350 (2012). 22238126 10.1002/pro.2019 PMC3375435

[b53] Wang Y. *et al.* The higher barrier of darunavir and tipranavir resistance for hiv-1 protease. Biochem. Bioph. Res. Co. 412 (2011). 10.1016/j.bbrc.2011.08.045 PMC3188455 21871444

[b54] Vargiu A. V. & Nikaido H. Multidrug binding properties of the acrb efflux pump characterized by molecular dynamics simulations. Proc. Natl. Acad. Sci. USA 109, 20637–20642 (2012). 23175790 10.1073/pnas.1218348109 PMC3528587

[b55] Asthana S., Shukla S., Ruggerone P. & Vargiu A. V. Molecular Mechanism of Viral Resistance to a Potent Non-nucleoside Inhibitor Unveiled by Molecular Simulations. Biochemistry 53, 6941–6953 (2014). 25338932 10.1021/bi500490z

[b56] Gohlke H., Kiel C. & Case D. A. Insights into Protein-Protein Binding by Binding Free Energy Calculation and Free Energy Decomposition for the Ras-Raf and Ras-RalGDS Complexes. Journal of Molecular Biology 330, 891–913 (2003). 12850155 10.1016/s0022-2836(03)00610-7

[b57] Case D. A. *et al.* amber 2015, University of California, San Francisco (2015).

[b58] Maier J. A. *et al.* ff14sb: Improving the accuracy of protein side chain and backbone parameters from ff99sb. J. Chem. Theory Comput. 11, 10.1021/acs.jctc.5b00255, 3696–3713 (2015). 26574453 PMC4821407

[b59] Joung I. S. & Cheatham T. E. III Determination of alkali and halide monovalent ion parameters for use in explicitly solvated biomolecular simulations. J. Phys. Chem. B 112, 9020–9041 (2008). 18593145 10.1021/jp8001614 PMC2652252

[b60] Laskowski R. A. & Swindells M. B. Ligplot+: multiple ligand-protein interaction diagrams for drug discovery. J. Chem. Inf. Model. 51, 2778–2786 (2011). 21919503 10.1021/ci200227u

