# Supplementary material for: HIV-1 Protease Dimerization Dynamics Reveals a Transient Druggable Binding Pocket at the Interface
Source: Sci Rep. 2015 Dec 22;5:18555. doi: 10.1038/srep18555 (PMC4686983; doi:10.1038/srep18555)
Supplement: Supplementary Information [file srep18555-s1.pdf]

Supporting Information:

HIV-1 Protease Dimerization Dynamics Reveals a Transient Druggable Binding Pocket at the Interface

Fabio Pietrucci,† Attilio Vittorio Vargiu,‡ and Agata Kranjc¶

† Sorbonne Universités, UPMC University Paris 6, CNRS - UMR 7590, IMPMC, F-75005 Paris, France
‡ Department of Physics, University of Cagliari, I-09042 Monserrato, Italy,
¶ School of Pharmaceutical Sciences, University of Geneva, CH-1211 Geneva, Switzerland

MM-GBSA binding free energy calculations

Molecular Dynamics of HIV protease adducts.

We performed molecular dynamics simulations for the following four complexes: HIV-TPV starting from the X-ray structure with PDB code 2O4P ((1), hereafter HIV_{TPV}^{Xray}); HIV-TPV starting from the best pose obtained by docking tipranavir in the new pocket (hereafter HIV_{TPV}^{nBP}); HIV-DRV starting from the X-ray structure with PDB code 4LL3 ((2), hereafter HIV_{DRV}^{Xray}); HIV-DRV starting from the best pose obtained by docking darunavir in the new pocket (hereafter HIV_{DRV}^{nBP}). Note that the structure of the HIV protease in 2O4P is actually a variant of the wt protein, bearing the single-point Q7K mutation (known to reduce autoproteolysis of the enzyme). We chose this experimental source due to the lack of any published structure of the wt HIV-1 protease in complex with TPV. Despite this mutation could in principle influence the binding of the ligand, we notice that: a) the site of mutation remains quite far from the inhibitor (the minimum distance between each couple of atoms of K7 and TPV is larger than 10 Å in the experimental structure and over the whole course of the simulation), and K7 points always toward the solvent, thus it is unlikely to be involved in direct or water-mediated interactions with TPV. This is confirmed by the analysis of per-residue contributions to the binding, showing that Q/K does not contribute to the stabilization of TPV (Table S1); b) the region of the protein around the site of mutation has virtually the same structure of the wt protein (the average RMSD considering the sequence from residue P1 to D30 is around 0.5 Å). For these reasons, we believe that, while the mutation might have an effect on the dynamics of the molecular recognition, it is unlikely that it will affect any of the properties we have calculated in this work, in particular the binding position and affinity.
Each complex was solvated with water within a truncated octahedral box with 16 Å cutoff, and 5 Chlorine ions were randomly inserted in the solvent in order to reach system neutrality. Each system contained about 70.000 atoms. All-atom simulations were performed with the AMBER14 package (3). As the purpose of this part of the work was to characterize the thermodynamics signatures of the new binding site compared to the experimental one, we performed a multi-step partially restrained dynamics as follows (4):

1. Structural relaxation in the presence of soft restraints (1 $kcal \cdot mol^{-1} \cdot Å^{-2}$) on all the non-hydrogenous atoms of the protein and the ligand. In the second and third steps, the restraints were kept only on backbone and Cα atoms, respectively, and on the non-

hydrogenous atoms of ligand. Finally, restraints were removed from the ligand and from a selection of residues having at least one atom within 4 Å from the ligand. In each step the structure of the solute from previous run was used as target for restraints, and up to 25.000 optimization steps were performed using the conjugate-gradients algorithm.

2. Next, annealing up to 340 K was performed in 2 ns, using the same setup as in the last step of the relaxation described at the previous point, and constant volume and temperature conditions (NVT ensemble). This was followed by quenching to 310K in 1 ns, and then by a 1-ns long equilibration with same setup as above, but in the NTP ensemble.

3. Finally, a partially restrained MD run of 5 ns, using the same setup as above was performed. Trajectories were saved every 50 ps.
A time step of 2 fs was used, and periodic boundary conditions were employed, and electrostatic interactions were treated using the particle-mesh-Ewald (PME) method, with a real-space cutoff of 9 Å and a grid spacing of 1 Å per grid point in each dimension. The van der Waals interactions were modeled with a Lennard-Jones potential, using also a cutoff of 9 Å. The simulations were performed in the NPT ensemble and the temperature was kept at 310 K by applying the Langevin thermostat with a collision frequency set to 5 ps^{-1}. The pressure was kept at 1.013 bar using a Berendsen barostat (5).
The force field parameters of both ligands with formal charge equal to zero were taken from the GAFF force field (6), and the missing ones were generated using the antechamber/parmchk2 modules of AMBER14. Namely, atomic restrained electrostatic potential (RESP) charges were derived using the antechamber tool of AMBER, after structural optimization at the b3lyp/6-31G(d,p) level performed with Gaussian09(7) in the presence of implicit solvent (PCM) in order to avoid overstabilization of intramolecular H-bonds. The parameters of the compounds investigated here are available upon request to the authors. The parm14SB force field was used to parametrize the protein, in conjunction with the TIP3P (8) model for water and the Joung&Cheatam modified parameters for ions (9).

<u>Post-processing of trajectories.</u>

The free energy of binding of the inhibitors to the protein was evaluated by means of the Molecular Mechanics – Generalized Born Surface Area (MM-GBSA) post-processing method (10, 11) using the MMPBSA.py tool of the AmberTools package (12).
According to the MM-GBSA theory (13, 14), the free energy of binding ΔG_{bind} is evaluated through the formula:

$$\Delta G_{bind} = G_{com} - \left(G_{rec} + G_{lig} \right).$$

G_{com}, G_{rec}, and G_{lig} are the absolute free energies of complex, receptor, and ligand, respectively, averaged over the equilibrium trajectory of the complex (single trajectory approach). According to these schemes, the free energy difference can be decomposed as:

$$\Delta G_{bind} = \Delta E_{MM} + \Delta G_{solv} - T\Delta S_{conf}$$

where ΔE_{MM} is the difference in the molecular mechanics energy (null in the single-trajectory approach, within which the structures of the apo-protein and the ligand are

extracted from the dynamics of the complex), ΔG_{solv} is the solvation free energy, and $T\Delta S_{conf}$ is the solute conformational entropy. The first two terms were calculated with the following equations:

$$\Delta E_{MM} = \Delta E_{bond} + \Delta E_{angle} + \Delta E_{torsion} + \Delta E_{vdw} + \Delta E_{elec}$$

$$\Delta G_{solv} = \Delta G_{solv,p} + \Delta G_{solv,np}$$

E_{MM} includes the molecular mechanics energy contributed by the bonded (E_{bond}, E_{angle}, and $E_{torsion}$) and non-bonded (E_{vdw} and E_{ele}, calculated with no cutoff) terms of the force field. ΔG_{solv} is the solvation free energy, which can be modeled as the sum of an electrostatic contribution ($\Delta G_{solv,p}$, evaluated using the MM-GBSA approach) and a non-polar one ($\Delta G_{solv,np} = \gamma \Delta_{SA} + b$, proportional to the difference in solvent-exposed surface area, Δ_{SA}).

In the MM-GBSA approach, the electrostatic solvation free energy was calculated using the implicit solvent model in Ref. (15, 16) (igb = 8 option in AMBER14) in combination with mbondi3 (17, 18) (for H, C, N, O, S elements) and intrinsic(19) radii. Partial charges were taken from the AMBER/GAFF force fields, and relative dielectric constants of 1 for solute and 78.4 for the solvent (0.1 M KCl water solution) were used. The non-polar contribution is approximated by the LCPO (20) method (20) implemented within the *sander* module of AMBER. In addition to being faster, the MM-GBSA approach furnishes an intrinsically easy way of decomposing the free energy of binding into contributions from single atoms and residues(21), which is alternative to the “alanine scanning” approach (4, 21-24).

Solvation free energies were calculated on the optimized poses and on 80 frames extracted from the last 4 ns of the partly restrained MD simulation. The solute conformational entropy contribution ($T\Delta S_{conf}$) is composed by a rototranslational term, calculated through classical statistical mechanics formulas, and by a vibrational term, which has been estimated here through normal-mode analysis using the *mmpbsa_py_nabnmode* module of AMBER14. Solute entropies were calculated, for each system, on the optimized poses and on 20 snapshots taken every 200 ps from the 4 ns of the MD trajectory.

	Opt				MD			
Residue	HIV_{TPV}^{Xray}	HIV_{TPV}^{nBP}	HIV_{DRV}^{Xray}	HIV_{DRV}^{nBP}	HIV_{TPV}^{Xray}	HIV_{TPV}^{nBP}	HIV_{DRV}^{Xray}	HIV_{DRV}^{nBP}
T4		-1.6				-1.4 (0.2)		**-0.3 (0.6)**
L5		-2.0		-1.5		**-1.9 (0.3)**		-1.0 (0.5)
R8			-1.1					
T26		-3.5		-1.4		**-2.4 (0.6)**		**-1.2 (0.8)**
G27			-1.8					
A28	-2.5		-1.9		-2.0 (0.5)			
D30			-3.0					
I47	-2.9				-2.3 (0.2)			
G49	-2.0				-1.8 (0.3)			
I50	-2.7		-2.2		-2.8 (0.7)		-2.9 (0.5)	
P81	-1.2				-1.4 (0.2)			
V82			-1.0					
I84	-1.8		-1.5		-1.8 (0.2)		-1.1 (0.1)	
R87	-2.1			-1.7	-3.2 (0.4)	**-0.9 (0.9)**		**-1.0 (0.7)**
T91								**-0.2 (0.1)**
L97		-1.4				**-2.1 (0.4)**		
I3’						**-0.5 (0.1)**		
L5’		-1.2		-1.1		**-0.4 (0.1)**		**-0.8 (0.3)**
R8’	-4.5				-4.1 (0.2)	-1.3 (0.3)		
P9’						**-0.2 (0.2)**		
L23’	-1.4							
T26’		-4.2		-2.1		-3.1 (1.0)		**-1.3 (0.8)**
G27’	-1.2		-2.2		-1.6 (0.4)			
A28’	-1.4		-4.2		-1.2 (0.3)		-3.6 (0.9)	
D29’			-1.4				-1.7 (0.5)	
G49’	-2.4				-2.9 (0.3)			
I50’	-4.8		-2.5		-5.1 (0.4)		-3.0 (0.6)	
P81’	-1.3							
I84’	-1.5		-1.8		-1.3 (0.2)		-2.1 (0.5)	
R87’		-1.6				**-1.5 (0.6)**		**0.0 (0.9)**
L90’		-1.1				-1.5 (0.3)		
T91’		-2.3				**-1.4 (0.5)**		**-0.7 (0.1)**
G94’		-1.3						
T96’				-1.0				**-1.0 (0.3)**
L97’		-2.4		-3.1		-1.3 (0.4)		**-3.0 (0.3)**

Table S1. Relevant per-residue contributions to ΔG_b calculated for the four complexes investigated in this work. Values are in kcal/mol, and standard deviations are in parentheses for the values extracted from the post-processing of the MD trajectories. Residues appearing in LigPlots at Figs. 6 and 7 are reported with the values of the free energy highlighted bold. Concerning the other residues, only those contributing more than 1 kcal/mol to stabilization of the complex are reported.

REFERENCES

1. **Muzammil S, Armstrong AA, Kang LW, Jakalian A, Bonneau PR, Schmelmer V, Amzel LM, Freire E.** 2007. Unique Thermodynamic Response of Tipranavir to Human Immunodeficiency Virus Type 1 Protease Drug Resistance Mutations. Journal of virology **81:**5144-5154.
2. **Kožíšek M, Lepšík M, Grantz Šašková K, Brynda J, Konvalinka J, Řezáčová P.** 2014. Thermodynamic and structural analysis of HIV protease resistance to darunavir – analysis of heavily mutated patient-derived HIV-1 proteases. Febs J. **281:**1834-1847.
3. **Case DA, J.T. Berryman, R.M. Betz, D.S. Cerutti, Cheatham III TE, T.A. Darden, R.E. Duke, T.J. Giese, H. Gohlke, A.W. Goetz, N. Homeyer, S. Izadi, P. Janowski, J. Kaus, A. Kovalenko, T.S. Lee, S. LeGrand, P. Li, T. Luchko, R. Luo, B. Madej, K.M. Merz, G. Monard, P. Needham, H. Nguyen, H.T. Nguyen, I. Omelyan, A. Onufriev, D.R. Roe, A. Roitberg, R. Salomon-Ferrer, C.L. Simmerling, W. Smith, J. Swails, R.C. Walker, J. Wang, R.M. Wolf, X. Wu, York DM, Kollman PA.** 2015. AMBER 2015. University of California, San Francisco.
4. **Vargiu AV, Nikaido H.** 2012. Multidrug binding properties of the AcrB efflux pump characterized by molecular dynamics simulations. Proc. Natl. Acad. Sci. U.S.A. **109:**20637-20642.
5. **Berendsen HJC, Postma JPM, Vangunsteren WF, Dinola A, Haak JR.** 1984. Molecular-Dynamics with Coupling to an External Bath. J. Chem. Phys. **81:**3684-3690.
6. **Wang J, Wolf RM, Caldwell JW, Kollman PA, Case DA.** 2004. Development and testing of a general amber force field. J. Comput. Chem. **25:**1157-1174.
7. **Frisch MJ, Trucks GW, Schlegel HB, Scuseria GE, Robb MA, Cheeseman JR, Scalmani G, Barone V, Mennucci B, Petersson GA, Nakatsuji H, Caricato M, Li X, Hratchian HP, Izmaylov AF, Bloino J, Zheng G, Sonnenberg JL, Hada M, Ehara M, Toyota K, Fukuda R, Hasegawa J, Ishida M, Nakajima T, Honda Y, Kitao O, Nakai H, Vreven T, Jr. JAM, Peralta JE, Ogliaro F, Bearpark M, Heyd JJ, Brothers E, Kudin KN, Staroverov VN, Kobayashi R, Normand J, Raghavachari K, Rendell A, Burant JC, Iyengar SS, Tomasi J, Cossi M, Rega N, Millam JM, Klene M, Knox JE, Cross JB, Bakken V, Adamo C, Jaramillo J, Gomperts R, Stratmann RE, Yazyev O, Austin AJ, Cammi R, Pomelli C, Ochterski JW, Martin RL, Morokuma K, Zakrzewski VG, Voth GA, Salvador P, Dannenberg JJ, Dapprich S, Daniels AD, Farkas Ö, Foresman JB, Ortiz JV, Cioslowski J, Fox DJ.** 2009. Gaussian 09, Revision A.1, Revision A.1 ed. Gaussian, Inc., Wallingford CT.
8. **Jorgensen WL, Chandrasekhar J, Madura JD, Impey RW, Klein ML.** 1983. COMPARISON OF SIMPLE POTENTIAL FUNCTIONS FOR SIMULATING LIQUID WATER. J Chem Phys **79:**926-935.
9. **Joung IS, Cheatham TE.** 2008. Determination of alkali and halide monovalent ion parameters for use in explicitly solvated biomolecular simulations. J. Phys. Chem. B **112:**9020-9041.
10. **Kollman Pa, Massova I, Reyes C, Kuhn B, Huo S, Chong L, Lee M, Lee T, Duan Y, Wang W, Donini O, Cieplak P, Srinivasan J, Case Da, Cheatham TE.** 2000. Calculating

structures and free energies of complex molecules: combining molecular mechanics and continuum models. Acc. Chem. Res. **33:**889-897.

11. **Srinivasan J, Cheatham III TE, Cieplak P, Kollman PA, Case DA.** 1998. Continuum Solvent Studies of the Stability of DNA, RNA, and Phosphoramidate-DNA Helices. J. Am. Chem. Soc. **120:**9401-9409.
12. **Miller BR, McGee TD, Swails JM, Homeyer N, Gohlke H, Roitberg AE.** 2012. MMPBSA.py: An Efficient Program for End-State Free Energy Calculations. J. Chem. Theory Comp. **8:**3314-3321.
13. **Kollman PA, Massova I, Reyes C, Kuhn B, Huo SH, Chong L, Lee M, Lee T, Duan Y, Wang W, Donini O, Cieplak P, Srinivasan J, Case DA, Cheatham TE.** 2000. Calculating structures and free energies of complex molecules: Combining molecular mechanics and continuum models. Acc. Chem. Res. **33:**889-897.
14. **Fogolari F, Brigo A, Molinari H.** 2002. The Poisson-Boltzmann equation for biomolecular electrostatics: a tool for structural biology. J. Mol. Recognit. **15:**377-392.
15. **Shang Y, Nguyen H, Wickstrom L, Okur A, Simmerling C.** 2011. Improving the description of salt bridge strength and geometry in a Generalized Born model. J. Mol. Graph. **29:**676-684.
16. **Nguyen H, Roe DR, Simmerling C.** 2013. Improved Generalized Born Solvent Model Parameters for Protein Simulations. J. Chem. Theory Comp. **9:**2020-2034.
17. **Onufriev A, Bashford D, Case DA.** 2004. Exploring protein native states and large-scale conformational changes with a modified generalized born model. Proteins **55:**383-394.
18. **Bondi A.** 1964. van der Waals Volumes and Radii. J. Phys. Chem. **68:**441-451.
19. **Tsui V, Case DA.** 2000. Molecular dynamics simulations of nucleic acids with a generalized born solvation model. J. Am. Chem. Soc. **122:**2489-2498.
20. **Weiser J, Shenkin PS, Still WC.** 1999. Approximate atomic surfaces from linear combinations of pairwise overlaps (LCPO). J. Comput. Chem. **20:**217-230.
21. **Gohlke H, Kiel C, Case DA.** 2003. Insights into Protein–Protein Binding by Binding Free Energy Calculation and Free Energy Decomposition for the Ras–Raf and Ras–RalGDS Complexes. J. Mol. Biol. **330:**891-913.
22. **Asthana S, Shukla S, Vargiu AV, Ceccarelli M, Ruggerone P, Paglietti G, Marongiu ME, Blois S, Giliberti G, La Colla P.** 2013. Different Molecular Mechanisms of Inhibition of Bovine Viral Diarrhea Virus and Hepatitis C Virus RNA-Dependent RNA Polymerases by a Novel Benzimidazole. Biochemistry **52:**3752-3764.
23. **Vargiu AV, Magistrato A.** 2012. Detecting DNA Mismatches with Metallo-Insertors: A Molecular Simulation Study. Inorganic chemistry **51:**2046-2057.
24. **Asthana S, Shukla S, Ruggerone P, Vargiu AV.** 2014. Molecular Mechanism of Viral Resistance to a Potent Non-nucleoside Inhibitor Unveiled by Molecular Simulations. Biochemistry **53:**6941-6953.
